# National Trends in Arrhythmias and Heart Failure Related Mortality in the United States From 1999 to 2023: A CDC Wonder Analysis

**DOI:** 10.1002/joa3.70222

**Published:** 2025-11-19

**Authors:** Saeed Aftab Khan, Arfa Ahmed Assad, Maria Qadri, Sabahat Ul Ain Munir Abbasi, Hira Saleem, Muhammad Saeed, Shafiq Ur Rahman, Tahir Nawaz, Aala Saleh, Hamza Ashraf

**Affiliations:** ^1^ Department of Medicine Allama Iqbal Medical College Lahore Pakistan; ^2^ Department of Medicine Jinnah Sindh Medical University Karachi Pakistan; ^3^ Department of Medicine D.G. Khan Medical College Dera Ghazi Khan Pakistan; ^4^ Department of Medicine Saidu Group of Teaching Hospital Swat Pakistan; ^5^ Faculty of Medicine Lebanese University Beirut Lebanon

**Keywords:** arrhythmias, cardiovascular diseases, CDC WONDER, heart failure, mortality

## Abstract

**Background:**

Arrhythmias are an important contributing cause of death in patients with heart failure (HF). Despite advancements in cardiovascular care, the burden of arrhythmia‐related mortality in HF patients remains a significant public health concern. This study aims to evaluate national trends in arrhythmia‐related mortality in HF patients from 1999 to 2023.

**Methods:**

Mortality data for patients with arrhythmias (ICD‐10 codes: I44.0–I49.9) and HF (ICD‐10 codes: I11.0, I13.0, I13.2, I50.0, I50.1, and I50.9) was obtained from the CDC WONDER Database. Age‐adjusted mortality rates (AAMRs) were calculated per 100 000 populations. Joinpoint regression was used to assess the annual percentage changes in AAMR.

**Results:**

Between 1999 and 2023, arrhythmias and HF were responsible for 1 428 953 deaths in the United States. A notable increase in arrhythmia‐related mortality among HF patients during 2018–2021 (APC = 5.77%) was observed. Sex‐based disparities revealed higher mortality (AAMR: 37.61) in males. Racial and ethnic disparities were evident, with non‐Hispanic American Indians showing the highest AAPC (2.95). State‐level variations indicated that Oregon reported the highest AAMR (47.106), while Nevada reported the lowest (16.74). Geographic analysis showed higher mortality rates in non‐metropolitan areas.

**Conclusion:**

The observed reduction in arrhythmia‐related mortality in HF patients over the last two decades suggests promising advancements in public health strategies and cardiovascular care. However, the persistent disparities across geographic, demographic, and healthcare access lines underscore the need for deeper investigation into the underlying causes of these trends.

## Graphical abstract

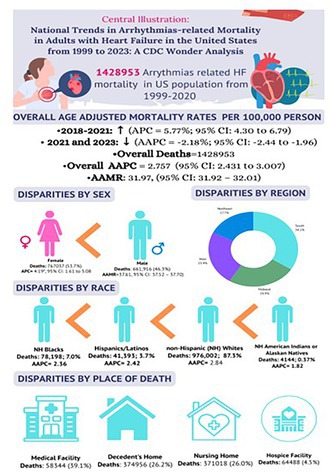

## 1 Introduction

Heart failure (HF) is an impaired heart function that cannot pump sufficient blood to the body [1]. Arrhythmias are irregular heart rhythms, and they can exacerbate HF symptoms and increase the risk of sudden cardiac death [2]. HF is a major health concern in the United States, affecting approximately 6.7 million adults, and contributing to significant morbidity and mortality. The prevalence is expected to reach 8.7 million by 2030, and approximately 11.14 million by 2050. HF mortality has increased proportionately contributing to 45% of cardiovascular deaths in the U.S. in 2021. The incidence is rising in younger adults as compared to older patients [3]. Advancement in the management of HF has significantly improved outcomes, but arrhythmias are still potential complications that are fatal to patients with HF [4]. Approximately 0.9 million deaths occurred in patients with HF having a concomitant arrhythmia in the U.S. from 1999 to 2020 [5].

In 2017, HF affected nearly 64.3 million people worldwide with a 1‐year mortality rate of 15%–30%, becoming the leading cause of death globally [6]. Between 1990 and 2021, the HF prevalence rate per 100 000 population increased from 125.5 to 148.1 [7]. Arrhythmias are a significant contributor to death in patients with HF worldwide, causing 30%–50% of deaths in this population [8]. It is estimated that the incidence and prevalence of atrial fibrillation (AF), which is the most common type of arrhythmias, will be doubled in the U.S. by 2030 [9]. The impact of HF extends beyond the individual level, as it imposes a potential economic burden on the healthcare system [10].

Trends and disparities in arrhythmia‐related mortality and HF‐related mortality among US adults have been discussed separately [11, 12], but their association remains unexplored. It is crucial to understand the temporal trends of arrhythmia‐related mortality in HF patients to formulate targeted prevention strategies and improve patient care. The Global Burden of Disease study has estimated the global, regional, and national burdens of HF associated with AF worldwide but the national trends among US adults need to be examined [13]. This study utilizes the Centers for Disease Control and Prevention's (CDC) WONDER (Wide‐ranging Online Data for Epidemiologic Research) database to examine national trends in arrhythmia‐related mortality in HF patients in the United States from 1999 to 2023. By analyzing this comprehensive dataset, our study aims to identify patterns and shifts in mortality rates over time, assess potential demographic disparities, and explore the impact of evolving regional variations. The findings of this study will help future research aimed at reducing arrhythmia‐related deaths, providing valuable insights for public health practitioners and policymakers, and improving the quality of life for individuals with HF.

## 2 Methods

### 2.1 Study Setting and Study Design

We retrospectively analyzed the mortality trends related to arrhythmia and heart failure (HF) in the United States from 1999 to 2023 using data retrieved from the CDC Wide‐Ranging Online Data for Epidemiologic Research (CDC WONDER) database, maintained by the National Center for Health Statistics (NCHS) [14]. The dataset comprises death certificate data from all 50 U.S. states and the District of Columbia, with annually updated records for the underlying and contributing causes of death as well as demographic details. Mortality cases were identified where arrhythmia and HF were recorded as either the underlying or a contributing cause of death. To ensure consistency and clarity, data were extracted using the Multiple Cause of Death Public Use Record and International Classification of Diseases, 10th Revision, Clinical Modification (ICD‐10) codes defining the “Overall Heart Failure‐related death” as deaths where any of the following ICD‐10 codes were listed: I11.0, I13.0, I13.2, I50.0, I50.1, and I50.9. “Overall Heart Failure and Arrhythmias‐related death” included all HF codes listed above in addition to arrhythmia‐related codes: I44.0‐I44.7, I45.0‐I45.6, I45.8, I45.9, I47.0, I47.1, I47.2, I47.9, I48, I 49.0‐I49.5, I49.8, I49.9. This broader classification reflects terminal arrhythmic complications frequently associated with progressive HF. These classification methods align with previous studies that have validated the use of ICD‐10 codes for reliably identifying specific causes of cardiovascular death in large national datasets [11, 15, 16, 17]. The study adhered to the Strengthening the Reporting of Observational Studies in Epidemiology (STROBE) guidelines for observational research [18]. Institutional review board approval was not required, as the study used publicly available, anonymous patient data.

### 2.2 Data Extraction

Extracted variables included: year of death, place of death (Medical facility, Decedent's Home, Hospice Facility, Nursing home/Long term care, Place of death unknown, other), sex (Male/Female), race/ethnicity (Hispanic, non‐Hispanic Asian or Pacific Islander, non‐Hispanic White, non‐Hispanic Black or African American, non‐Hispanic American Indian or Alaska Native), urbanization (Metropolitan, non‐Metropolitan), and geographic region (Northeast, Midwest, South, and West based on U.S. Census Bureau definitions). Urbanization was classified using the National Center for Health Statistics Urban–Rural Classification Scheme (2013) [19], which categorized counties based on population size. Geographic regions were categorized according to the U.S. Census Bureau definitions into Northeast, Midwest, South, and West [20]. Data for race and urbanization was available until 2020, while all other demographic variables covered the full study period (1999–2023).

### 2.3 Statistical Analysis

Crude mortality rates (CMRs) and age‐adjusted mortality rates (AAMRs) per 100 000 populations were calculated for arrhythmia and HF‐related deaths. CMRs were derived by dividing the total number of deaths by the corresponding U.S. population each year. AAMRs were standardized to the 2000 U.S. population to allow for comparisons across time and demographic subgroups, with 95% confidence intervals (CIs) [21]. Temporal trends in AAMRs were analyzed using Joinpoint Regression (version 5.1.0, National Cancer Institute) to estimate annual percentage changes (APCs) and their 95% CIs [22]. Log‐linear regression models were applied to assess any significant shifts in mortality trends over time. An increase or decrease in APC was determined if its slope representing the change in mortality significantly differed from zero, as determined by 2‐tailed *t*‐test; otherwise, the trend was considered stable. To check the validity and strength of our findings, we conducted sensitivity analyses comparing mortality trends when arrhythmia and HF were listed solely as the underlying cause of death versus when they appeared as either the underlying or a contributing cause. A *p*‐value < 0.05 indicates statistical significance.

## 3 Results

### 3.1 Overall

**FIGURE 1:**
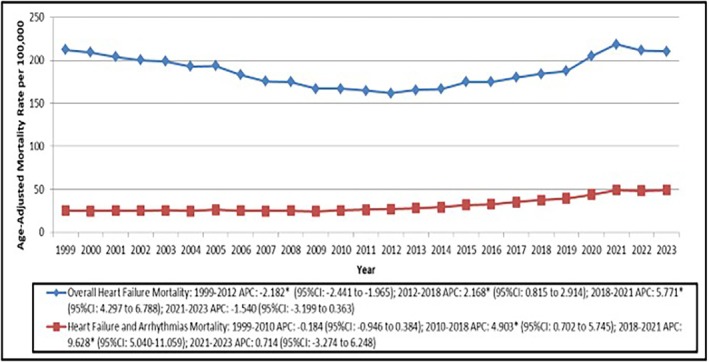
Trends in arrhythmias age‐adjusted mortality rates per 100 000 in adults with heart failure in the United States, overall, and stratified by gender, from 1999 to 2023.

Arrhythmia‐related mortality in HF patients was the cause of death of 1 428 953 people in the United States from 1999 to 2023 (AAMR: 31.97 per 100 000 population, 95% CI: 31.92–32.01 per 100 000; Figure 1, Table 1). Stratified by place of death, out of these 1 428 953 deaths, 558 344 (39.1%) deaths took place in medical facilities, 374 956 (26.2%) in decedent's home, 371 018 (26.0%) in nursing homes, and lastly 64 488 (4.5%) in hospice facilities (Table 1). The age‐adjusted mortality rates for deaths related to HF showed a statistically significant decrease in the period between 2021 and 2023 (AAPC = −2.18*, 95% CI = −2.44 to −1.96). However, the period of 2018–2021 immediately preceding this interval displayed a statistically significant increase in the age‐adjusted mortality rate (APC = 5.77*, 95% CI = 4.30 to 6.79).

**TABLE 1:** Arrhythmias related overall death and AAPC of age‐adjusted mortality rates in adults with heart failure in the United States from 1999 to 2023.

Variable	Total deaths (*n*)	AAPC (95% confidence interval)
Overall	1 428 953 (100%)	2.757* (2.431–3.007)
Gender		
Male	661 916 (46.3%)	3.030* (2.858–3.192)
Female	767 037 (53.7%)	2.421* (2.180–2.664)
Census region		
Northeast	252 453 (17.7%)	2.356* (2.1912–2.524)
Midwest	355 168 (24.9%)	2.658* (2.434–2.900)
South	487 451 (34.1%)	3.002* (2.831–3.197)
West	333 881 (23.4)	2.859* (2.648–3.112)
Race/ethnicity^a^		
NH American Indian or Alaska Native	4144 (0.37%)	2.951* (2.056–4.208)
NH Asian or Pacific Islander	17 735 (1.59%)	1.825* (1.449–2.302)
NH Black or African American	78 198 (7.0%)	2.360* (2.061–2.646)
NH White	976 002 (87.3%)	2.843* (2.634–3.065)
Hispanic	41 393 (3.7%)	2.423* (1.965–2.877)
Urbanization^a^		
Metropolitan	884 540 (79.0)	2.529* (2.258–2.764)
Non metropolitan	234 925 (21.0%)	2.977* (2.799–3.160)
Place of death		
Medical facility	558 344 (39.1%)	—
Decedent's home	374 956 (26.2%)	—
Hospice facility	64 488 (4.5%)	—
Nursing home/long‐term care facility	371 018 (26.0%)	—
Other	58 167 (4.1%)	—
Place of death unknown	1936 (0.14%)	

^a^ Total Deaths and AAPC is from 1999 to 2020.

* Indicates that AAPC is significantly different from zero at the alpha = 0.05.

Arrhythmia‐related mortality in HF also showed the greatest increase in the period between 2018 and 2021, with an APC of 9.63* (95% CI = 5.04–11.06; Table S1). This was followed by a stable trend in the period of 2021–2023, with an AAPC of 0.71 (95% CI = −3.27 to 6.25; Table S1).

### 3.2 Trends by Sex

**FIGURE 2:**
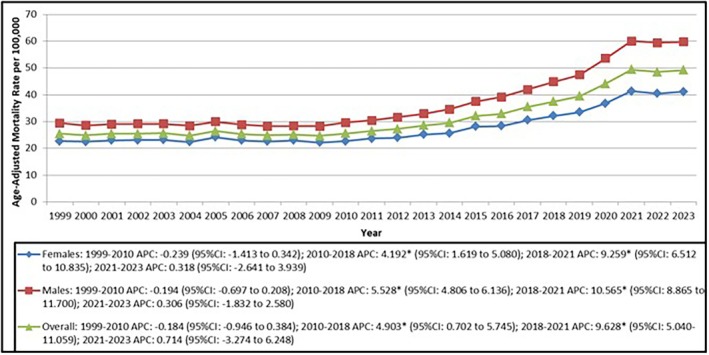
Trends in overall heart failure vs. heart failure and arrhythmias age‐adjusted mortality rates per 100 000 in adults in the United States from 1999 to 2023.

The overall arrhythmia‐related mortality in HF patients was higher in females, with a total of 767 037 (53.7%) deaths in the period between 1999 and 2023. Males, however, contributed to a total of 661 916 (46.3%) deaths (Figure 2, Table 1, Table S2). Contrary to the aforementioned statistics, males showed greater AAMR with a value of 37.61 per 100 000 population (95% CI: 37.52–37.70; Tables S2 and S3) Additionally, males displayed a higher APC in the periods of 2010–2018 (APC = 5.53*, 95% CI = 4.81 to 6.14) and 2018–2021 (APC = 10.57*, 95% CI = 8.87 to 11.70; Figure 2, Table S1) respectively as opposed to females in the same periods (APC = 4.19*, 95% CI: 1.61 to 5.08; APC = 9.26*; 95% CI: 6.51 to 10.83; Figure 2, Table S1) respectively. However, it must be mentioned that in the period between 2021 and 2023, the annual percent change in arrhythmias‐related mortality causes was higher among female patients (APC = 0.318, 95% CI: −2.64 to 3.93; Figure 2, Table S1). Overall, the highest age‐adjusted mortality rates for both male and female patients were noted in 2021, with a value of 41.4 (41–41.8) for females and 60 (59.5–60.5) for males (Figure 2).

### 3.3 Trends by Race/Ethnicity

**FIGURE 3:**
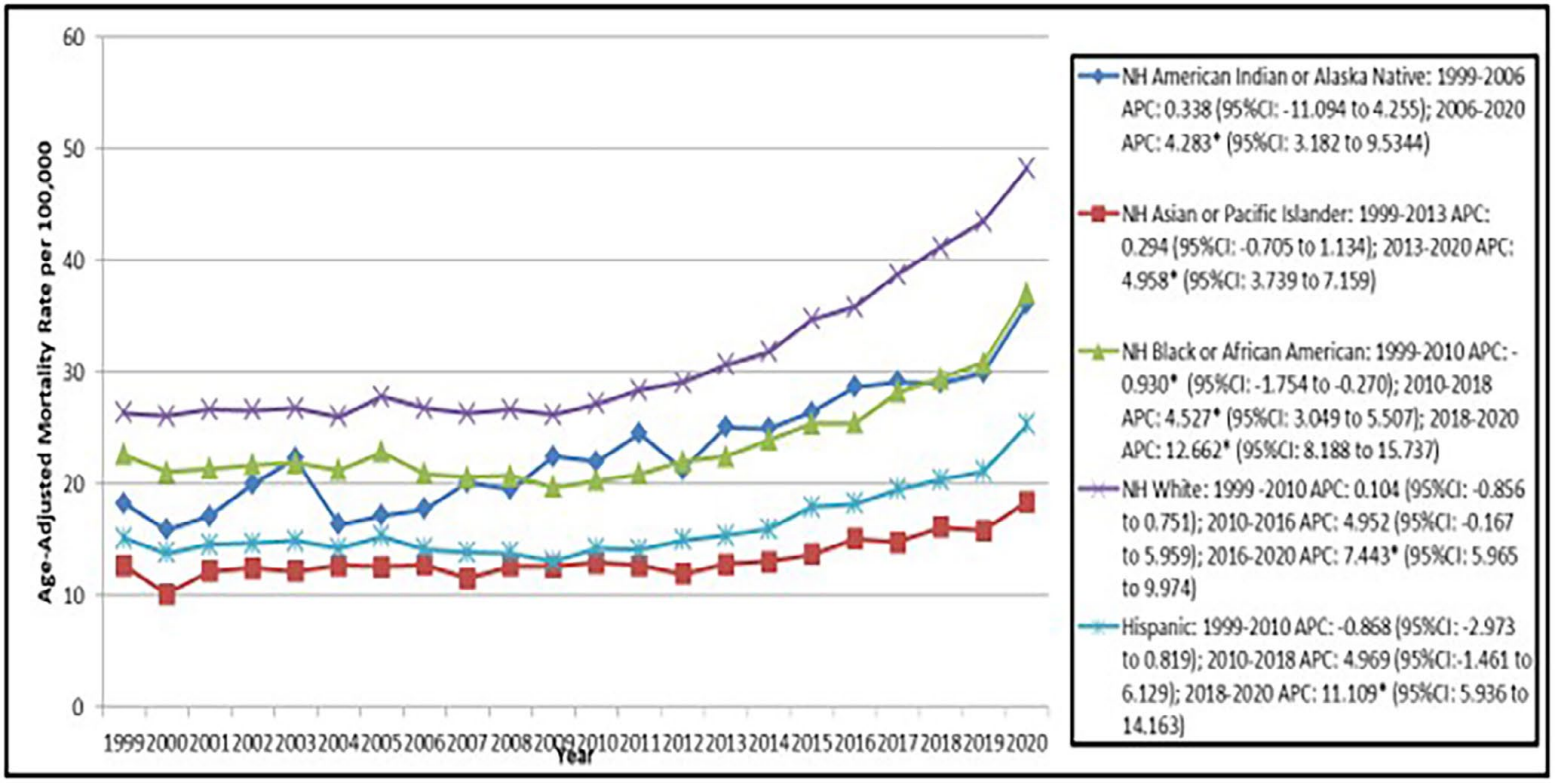
Trends in arrhythmias age‐adjusted mortality rates per 100 000 adults with heart failure in the United States, stratified by race, from 1999 to 2020.

When stratified by race/ethnicity, non‐Hispanic (NH) Whites (976 002; 87.3%) reported the highest number of deaths, followed by NH Blacks (78 198; 7.0%), Hispanics/Latinos (41 393; 3.7%), NH Asians (17 735; 1.59%), and NH American Indians or Alaskan Natives (4144; 0.37%; Figure 3, Table 1). The greatest AAPC, however, was reported in NH American Indians or Alaska Natives (2.95, 95% CI: 2.06 to 4.21). The second highest AAPC was reported in NH Whites (AAPC = 2.84*, 95% CI: 2.63 to 3.06), followed by Hispanics (AAPC = 2.42*, 95% CI: 1.97 to 2.88), NH Blacks (AAPC = 2.36*, 95% CI: 2.06 to 2.65) and NH Asians (AAPC = 1.82*, 95% CI: 1.45 to 2.30; Figure 3, Tables S2 and S4).

Analyzing the specific time segments of the aforementioned trends, the period between 2018 and 2020 caused a massive increase in the APC among NH blacks with a statistically significant value of 12.66* (95% CI: 8.19 to 15.74; Figure 3, Table S1). A similar increase for the same time period was observed for the Hispanic population, with an APC of 11.11* (95% CI: 5.93 to 14.16; Figure 3, Table S1). Among the NH white populations, the AAPC displayed a statistically significant value of 7.44* (95% CI: 5.97 to 9.97; Figure 3, Table S1) for the period between 2016 and 2020. Similar positive values of APC were noted for NH Asian and NH American Indian populations in the periods of 2013–2020 and 2006–2021 (AAPC = 4.96*, 95% CI: 3.74 to 7.16; AAPC = 4.28*, 95% CI: 3.18 to 9.534; Figure 3, Table S1) respectively.

### 3.4 Trends by Geographical Patterns

#### 3.4.1 Urban–Rural

**FIGURE 4:**
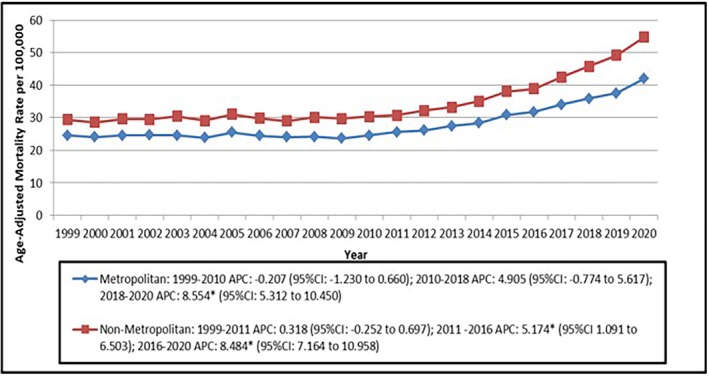
Trends in arrhythmias age‐adjusted mortality rates per 100 000 adults with heart failure in the United States, stratified by urban–rural classification, from 1999 to 2020.

Although the metropolitan areas comprised 79% of total deaths (884540), the respective AAMR was lower (AAMR: 2.53, 95% CI: 2.26 to 2.76) as compared to non‐metropolitan areas (AAMR: 2.98, 95% CI: 2.80 to 3.16; Figure 4, Table 1). Metropolitan areas showed the largest statistically significant increase in AAMR in the period between 2018 and 2020 (8.55; 95% CI: 5.31 to 10.45). Non‐metropolitan areas, on the other hand, showed a significant increase in the AAMR in the period between 2016 and 2020 (8.48, 95% CI: 7.16 to 10.96; Figure 4, Table S5).

#### 3.4.2 Census Region and Statewide

The mortality trends of the various census regions displayed significant variations, with the South having the highest overall number of deaths (487 451; 34.1%), followed by the Midwest and West, with AAMRs of 355 168 (24.9%) and 333 881 (23.4%) respectively. The Northeast had the lowest number of deaths observed (AAMR = 252 453; 17.7%; Table 1, Figure S6). The AAMRS for the census regions of the Midwest and South showed an increasing trend for the 2021–2023 period, but these results were statistically insignificant. For the same periods, the West and the Northeast displayed decreasing AAMRs (AAPC = −0.369, 95% CI: −2.69 to 2.58; AAPC = −0.30, 95% CI: −2.23 to 1.74; Figure S1, Table S1). Upon conducting the analysis based on state‐related AAMRs per 100 000 population, we deduced that the highest AAMRs were displayed by Oregon with values of 47.106 (95% CI: 46.506–47.706) in the period 1999–2020 and 88.541 (95% CI: 87.102–89.98) in the period 2018–2023 (Figure S2, Table S7).

This was followed by Minnesota (AAMR = 40.34, 95% CI: 39.86–40.82) in 1999–2020 and (AAMR = 76.56, 95% CI: 75.40–77.72) in 2018–2023 (Figure S2, Table S2). Conversely, states like Nevada (AAMR: 16.74, 95% CI: 16.23–17.25) and Florida (AAMR: 17.83, 95% CI: 17.68–17.98) exhibited the lowest AAMRs (Figure S2, Tables S2 and S8). Nearly half of the deaths took place in medical facilities (Table S9).

## 4 Discussion

**FIGURE 5:**
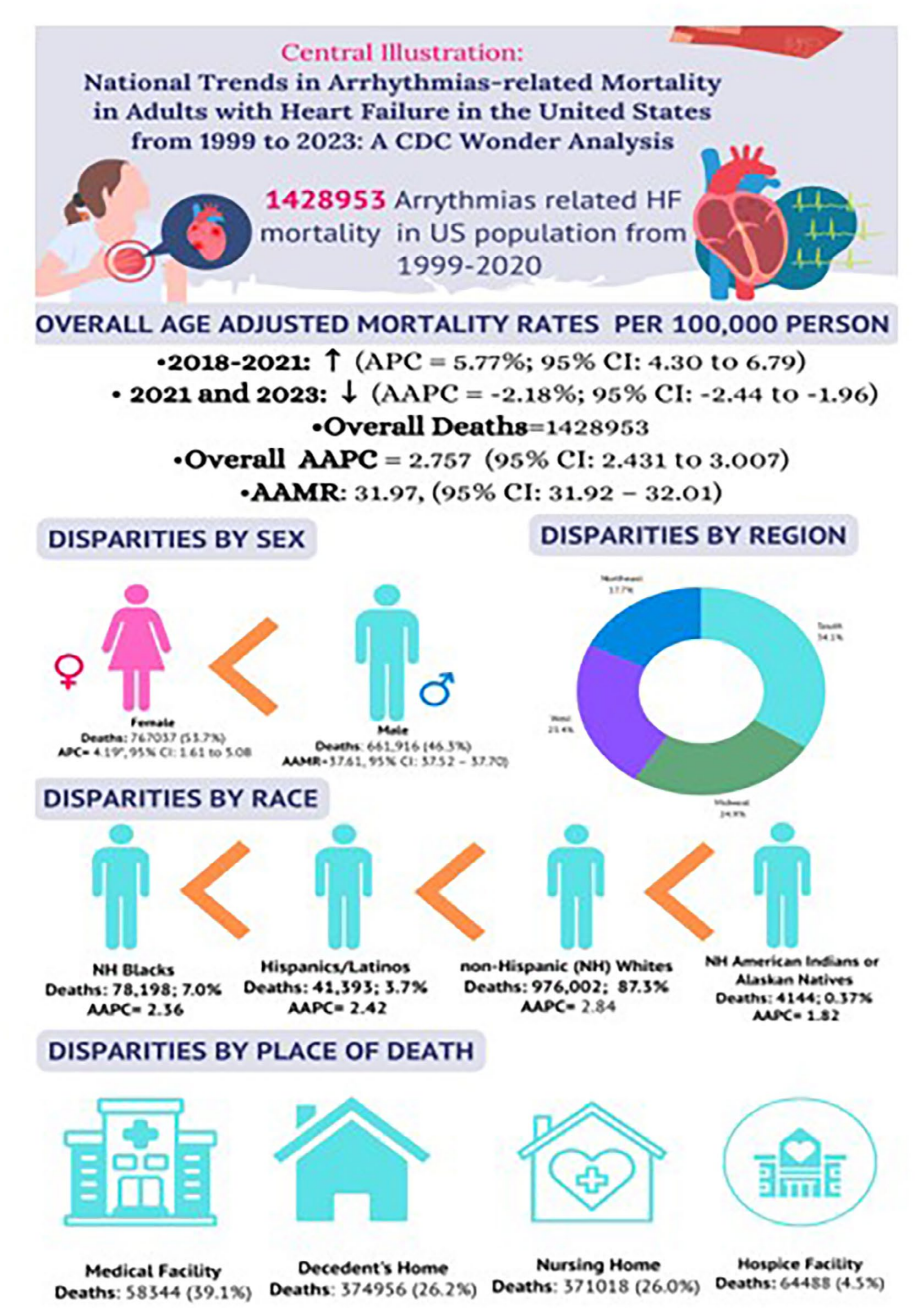
Central illustration.

HF is a primary cause of cardiovascular morbidity and mortality globally, with arrhythmias contributing considerably to unfavorable outcomes [23]. Our study revealed that arrhythmia‐related mortality in HF patients resulted in 1 428 953 deaths, with an age‐adjusted mortality rate (AAMR) of 31.97 per 100 000 populations, according to our analysis of U.S. mortality data from 1999 to 2023. These results are consistent with earlier research showing that 30%–50% of mortality from HF is caused by malignant arrhythmias and sudden cardiac death (SCD) [24]. Our study's temporal trends demonstrate dynamic changes in arrhythmia‐related mortality that are impacted by changing treatment approaches, shifting demographics, and healthcare inequities (Figure 5).

The sudden increase in HF and deaths related to arrhythmias between 2018 and 2021 aligns with the COVID‐19 pandemic, which interrupted cardiovascular treatment and worsened HF decompensation [25]. Furthermore, delayed hospitalization and reduced access to implantable cardioverter‐defibrillators (ICDs) might have led to increased mortality rates [26]. The subsequent decrease starting in 2021 might be due to the return of regular cardiovascular care, better adherence to guidelines for GDMT, and improvements in the treatment of arrhythmias, such as the increased use of implanted cardioverter‐defibrillators (ICDs) and catheter ablation techniques [27, 28].

Additionally, gender differences were apparent in the trends of mortality. Despite more deaths in females overall, males had higher rates of mortality. Particularly significant was the surge in mortality rates for males from 2010 to 2021, in contrast to females during the same timeframe. Our research shows that women represented 53.7% of arrhythmia‐related mortality in HF patients, while men displayed a higher age‐adjusted mortality rate (37.61 versus 31.97 per 100 000). This finding is consistent with the previous literature which indicates that women experiencing HF might have a greater tendency to show symptoms of heart failure with preserved ejection fraction (HFpEF), which is linked to a decreased rate of arrhythmia‐related mortality when compared to heart failure with reduced ejection fraction (HFrEF) [28, 29].

NH Whites recorded the greatest number of deaths in absolute terms (87.3%), while NH American Indians/Alaska Natives showed the highest (AAPC) at 2.95%, closely followed by NH Whites at 2.84%. During the period from 2018 to 2020, NH Blacks and Hispanics saw significant increases in death rates, with (APC) of 12.66% and 11.11%, respectively. These results are consistent with previous studies showing that structural inequities, restricted access to GDMT, and increased hospitalization rates for HF led to poorer outcomes in minority groups. The COVID‐19 pandemic intensified these inequalities, with Black and Hispanic communities experiencing higher rates of infection and diminished access to HF treatment [29, 30]. Past studies have indicated that Black NH face a greater prevalence of HF risk factors, such as hypertension and chronic kidney disease, which increase their susceptibility to arrhythmias and lead to poorer outcomes in HF [31]. Moreover, the South had the highest mortality burden and the largest share of all deaths, which was in line with regional trends of higher incidence of cardiovascular risk factors (such as obesity, diabetes, and hypertension) [32].

Furthermore, our study highlighted that the discrepancies in healthcare access and specialist cardiac care in rural communities were highlighted by the higher mortality rates in non‐metropolitan regions, even though metropolitan areas had a higher absolute number of fatalities [32]. According to earlier research, rural patients are less likely to have access to electrophysiology specialists and cutting‐edge arrhythmia treatments, which raises fatality rates.

According to the state‐level analysis, our study showed that Oregon had the highest death rates throughout both the earlier and later research periods, with Minnesota coming in second. These developments may be caused by a variety of factors, including access to sophisticated cardiovascular care, state‐level healthcare legislation, and demographic changes. Nevada and Florida, on the other hand, had the lowest death rates, which might be due to variations in healthcare systems and population composition at the state level [33].

### 4.1 Limitations

Initially, there was a decrease in mortality from 1999 to 2009, which then shifted to a significant increase from 2009 to 2023, hitting a peak during the COVID‐19 pandemic in 2021. A slight decline occurred from 2021 to 2023, resulting in the 2023 age‐adjusted mortality rate (AAMR) reaching 137.3 per 100 000, marking a 23% rise since 1999 [11]. Trends HF showed a dual challenge: certain populations (like Medicare beneficiaries) experienced a slight decrease in HF incidence, while prevalence climbed to 6.7 million cases in 2023, with projections suggesting it will reach 8.5 million by 2030. HF‐related mortality increased sharply after 2010, worsened by the disruptions caused by the pandemic [11]. Our analysis of racial and urban–rural disparities was concluded in 2020 because of structural changes in federal data systems. The CDC's National Center for Health Statistics (NCHS) introduced a new Urban–Rural Classification Scheme in 2023, which significantly altered the definitions of metropolitan areas and reclassified 12% of U.S. counties making pre‐2023 data incompatible for longitudinal studies [34]. Simultaneously, the CDC stopped providing bridged‐race population estimates after 2020, moving away from the 1997 OMB standards in favor of more detailed race/ethnicity categories that prevent direct trend comparisons [35]. These methodological changes necessitated setting a 2020 cut‐off to avoid inconsistencies in categorization. Non‐Hispanic Whites exhibited the highest mortality rates due to arrhythmias. Among HF patients, Black women faced a 23.9% lifetime risk, which is notably higher than the 13.4% risk for White women. In terms of age‐related risks, individuals aged 65 and older constituted the majority of HF hospitalizations. The 10‐year risk for HF surged from 1.0% to 3.0% between 1999 and 2015, largely influenced by an aging population and cardiometabolic conditions [3]. Moreover the highest mortality rates from arrhythmia were found in the Midwest, with Oregon having the top rates among states. HF mortality was concentrated in the Appalachian region and the Southern “stroke belt.” Nonmetropolitan areas exhibited AAMRs for arrhythmia that were 15%–20% higher than those in urban locations, highlighting a lack of access to electrophysiology services and delays in emergency care. In terms of HF subtypes, HF with preserved ejection fraction (HFpEF) now represents over 65% of all new HF cases. The lifetime risk of HFpEF has increased to 19.3%, surpassing that of HFrEF at 11.4%. Between 1990 and 2014, factors such as obesity, hypertension, and diabetes elevated the risk of HF by 24%–62%. For arrhythmias, acute triggers related to COVID‐19, like myocarditis and QT‐prolonging medications, contributed to the spike in mortality observed in 2021 [11, 34]. The primary trend analysis was conducted up to 2020, with post‐2020 data presented as exploratory; therefore, any conclusions drawn from the latter should be interpreted with caution.

## 5 Conclusion

Hence, the findings of our study show notable geographic, demographic, and temporal changes in the mortality rate from arrhythmias in patients with HF. A stable trend in mortality was observed till 2010 followed by a significant rise in mortality. The enduring racial, gender, and geographic inequalities highlight the necessity of focused public health initiatives to provide fair access to evidence‐based cardiovascular treatment. The underlying mechanisms causing these increases should be further investigated in future studies, and treatments to reduce disparities in arrhythmia‐related mortality in HF patients should be assessed.

## Author Contributions

**Saeed Aftab Khan and Arfa Ahmed Assad:** conceptualization, data curation, and formal analysis. **Maria Qadri and Sabahat Ul Ain Munir Abbasi:** methodology. **Hamza Ashraf:** project administration and visualization. **Saeed Aftab Khan and Hamza Ashraf:** validation. **Saeed Aftab Khan, Arfa Ahmed Assad, Maria Qadri, Sabahat Ul Ain Munir Abbasi, Hira Saleem, Muhammad Saeed, Tahir Nawaz, Aala Saleh, and Hamza Ashraf:** writing – original draft. **Saeed Aftab Khan, Arfa Ahmed Assad, Maria Qadri, Sabahat Ul Ain Munir Abbasi, Hira Saleem, Muhammad Saeed, Shafiq Ur Rahman, Tahir Nawaz, Aala Saleh, and Hamza Ashraf:** writing – review and editing.

## Conflicts of Interest

The authors declare no conflicts of interest.

## Supporting information


**Data S1:** joa370222‐sup‐0001‐Supinfo.docx.
