# Supplementary material for: National Trends in Arrhythmias and Heart Failure Related Mortality in the United States From 1999 to 2023: A CDC Wonder Analysis
Source: J Arrhythm. 2025 Nov 19;41(6):e70222. doi: 10.1002/joa3.70222 (PMC12628278; doi:10.1002/joa3.70222)
Supplement: Supplementary file 1 — Data S1: joa370222‐sup‐0001‐Supinfo.docx. [file JOA3-41-e70222-s001.docx]

**SUPPLEMENTARY MATERIAL**

**Supplementary Table 1: Annual Percentage Change of Arrhythmias-related Age-Adjusted Mortality Rates per 100,000 in Adults with Heart Failure in the United States, 1999 to 2023**

| Year Interval | APC (95% Confidence Interval) |
| --- | --- |
| Overall Heart Failure | |
| 1999-2012 | -2.182* (-2.441 to -1.965) |
| 2012-2018 | 2.168* (0.815 to 2.914) |
| 2018-2021 | 5.771* (95%CI: 4.297 to 6.788) |
| 2021-2023 | -1.540 (95%CI: -3.199 to 0.363) |
| Overall Heart Failure and Arrhythmias | |
| 1999-2010 | -0.184 (-0.946 to 0.384) |
| 2010-2018 | 4.903* ( 0.702 to 5.745) |
| 2018-2021 | 9.628* (5.040-11.059) |
| 2021-2023 | 0.714 (95%CI: -3.274 to 6.248) |
| Females | |
| 1999-2010 | -0.239 (-1.413 to 0.342) |
| 2010-2018 | 4.192* (95%CI: 1.619 to 5.080) |
| 2018-2021 | 9.259* (95%CI: 6.512 to 10.835) |
| 2021-2023 | 0.318 (95%CI: -2.641 to 3.939) |
| Males | |
| 1999-2010 | -0.194 (-0.697 to 0.208) |
| 2010-2018 | 5.528* (4.806 to 6.136) |
| 2018-2021 | 10.565* (8.865 to 11.700) |
| 2021-2023 | 0.306 (-1.832 to 2.580) |
| Northeast | |
| 1999-2009 | -0.524* (-1.165 to -0.017) |
| 2009-2018 | 4.116* (3.290 to 4.700) |
| 2018-2021 | 8.847* (7.114 to 9.964) |
| 2021-2023 | -0.301 (-2.238 to 1.748) |
| Midwest | |
| 1999-2010 | -0.530 (-2.001 to 0.140) |
| 2010-2016 | 4.510* (0.576 to 6.342) |
| 2016-2021 | 8.597* (7.340 to 11.286) |
| 2021-2023 | 0.553 (-2.277 to 3.873) |
| South | |
| 1999-2011 | -0.138 (-0.821 to 0.339) |
| 2011-2018 | 5.823* (2.620 to 6.877) |
| 2018-2021 | 10.523* (8.382 to 11.947) |
| 2021-2023 | 1.518 (-0.815 to 4.750) |
| West | |
| 1999-2012 | 1.069 (-0.169 to 1.764) |
| 2012-2018 | 5.017* (0.794 to 6.119) |
| 2018-2021 | 8.763* (6.524 to 10.379) |
| 2021-2023 | -0.369 (-2.692 to 2.581) |
| Metropolitan**^a^** | |
| 1999-2010 | -0.207 (-1.230 to 0.660) |
| 2010-2018 | 4.905 (-0.774 to 5.617) |
| 2018-2020 | 8.554* (5.312 to 10.450) |
| Non-Metropolitan**^a^** | |
| 1999-2011 | 0.318 (-0.252 to 0.697) |
| 2011-2016 | 5.174* (1.091 to 6.503); |
| 2016-2020 | 8.484* (7.164 to 10.958) |
| NH American Indian or Alaska Native**^a^** | |
| 1999-2006 | 0.338 (-11.094 to 4.255) |
| 2006-2021 | 4.283* (3.182 to 9.5344) |
| NH Asian or Pacific Islander**^a^** | |
| 1999-2013 | 0.294 (-0.705 to 1.134) |
| 2013-2020 | 4.958* (3.739 to 7.159) |
| NH Black or African American**^a^** | |
| 1999-2010 | -0.930* (-1.754 to -0.270) |
| 2010-2018 | 4.527* (3.049 to 5.507) |
| 2018-2020 | 12.662* (8.188 to 15.737) |
| NH White**^a^** | |
| 1999-2010 | 0.104 (-0.856 to 0.751) |
| 2010-2016 | 4.952 (-0.167 to 5.959) |
| 2016-2020 | 7.443* (5.965 to 9.974) |
| Hispanics**^a^** | |
| 1999-2010 | -0.868 (-2.973 to 0.819) |
| 2010-2018 | 4.969 (-1.461 to 6.129) |
| 2018-2020 | 11.109* (5.936 to 14.163) |

a- APC and AAMR values are calculated from 1999 to 2020

*indicates that APC is significantly different from zero at the alpha = 0.05

NH = Non-Hispanic

**Supplementary Table 2: Arrhythmias-related Deaths in Adults with Heart Failure, Stratified by Gender, in the United States, 1999 to 2023**

|  | Deaths | | | | | | | |  |
| --- | --- | --- | --- | --- | --- | --- | --- | --- | --- |
| Year | Overall | Females | Males | NH American Indians or Alaska Native | NH Asian or Pacific Islander | NH Black or African Americans | NH White | Hispanic | Total Population |
| 1999 | 35292 | 20530 | 14762 | 90 | 331 | 2531 | 31391 | 869 | 1.4E+08 |
| 2000 | 35080 | 20609 | 14471 | 82 | 282 | 2372 | 31422 | 839 | 1.42E+08 |
| 2001 | 36457 | 21364 | 15093 | 88 | 377 | 2439 | 32537 | 938 | 1.45E+08 |
| 2002 | 37006 | 21600 | 15406 | 106 | 407 | 2540 | 32864 | 989 | 1.47E+08 |
| 2003 | 37798 | 22072 | 15726 | 121 | 417 | 2580 | 33532 | 1068 | 1.49E+08 |
| 2004 | 37181 | 21544 | 15637 | 97 | 463 | 2560 | 32949 | 1068 | 1.51E+08 |
| 2005 | 40534 | 23677 | 16857 | 104 | 503 | 2815 | 35848 | 1207 | 1.53E+08 |
| 2006 | 39763 | 22998 | 16765 | 112 | 538 | 2651 | 35235 | 1179 | 1.56E+08 |
| 2007 | 39863 | 22998 | 16865 | 123 | 519 | 2673 | 35304 | 1216 | 1.58E+08 |
| 2008 | 41242 | 23848 | 17394 | 125 | 594 | 2766 | 36416 | 1281 | 1.6E+08 |
| 2009 | 41282 | 23440 | 17842 | 152 | 633 | 2720 | 36431 | 1294 | 1.61E+08 |
| 2010 | 43455 | 24325 | 19130 | 154 | 678 | 2857 | 38266 | 1456 | 1.63E+08 |
| 2011 | 46542 | 26133 | 20409 | 178 | 739 | 3043 | 40951 | 1583 | 1.65E+08 |
| 2012 | 48880 | 26997 | 21883 | 172 | 747 | 3335 | 42749 | 1796 | 1.67E+08 |
| 2013 | 52466 | 28828 | 23638 | 219 | 874 | 3575 | 45755 | 1948 | 1.68E+08 |
| 2014 | 55551 | 29925 | 25626 | 222 | 958 | 3897 | 48174 | 2172 | 1.7E+08 |
| 2015 | 61791 | 33208 | 28583 | 260 | 1077 | 4286 | 53373 | 2603 | 1.72E+08 |
| 2016 | 64684 | 33972 | 30712 | 291 | 1250 | 4474 | 55722 | 2794 | 1.74E+08 |
| 2017 | 71130 | 37239 | 33891 | 303 | 1314 | 5141 | 61087 | 3147 | 1.76E+08 |
| 2018 | 77103 | 39866 | 37237 | 338 | 1521 | 5543 | 66094 | 3474 | 1.78E+08 |
| 2019 | 82505 | 42061 | 40444 | 353 | 1574 | 5965 | 70729 | 3755 | 1.79E+08 |
| 2020 | 93860 | 46799 | 47061 | 454 | 1939 | 7435 | 79173 | 4717 | 1.81E+08 |
| 2021 | 99916 | 49294 | 50622 | - | - | - | - | - | 1.83E+08 |
| 2022 | 104345 | 51982 | 52363 | - | - | - | - | - | 1.84E+08 |
| 2023 | 105227 | 51728 | 53499 | - | - | - | - | - | 1.86E+08 |

**Supplementary Table 3: Arrhythmias-related Age-Adjusted Mortality Rates per 100,000 in Adults with Heart Failure, Stratified by Gender, in the United States, 1999 to 2023**

|  | **Age-adjusted Mortality Rate (95% Confidence interval)** | |
| --- | --- | --- |
| **Year** | **Females** | **Males** |
| 1999 | 22.7 (22.4 - 23) | 29.4 (28.9 - 29.9) |
| 2000 | 22.5 (22.2 - 22.8) | 28.5 (28 - 29) |
| 2001 | 23 (22.7 - 23.3) | 29 (28.6 - 29.5) |
| 2002 | 23.1 (22.8 - 23.4) | 29.1 (28.6 - 29.6) |
| 2003 | 23.2 (22.9 - 23.5) | 29.1 (28.6 - 29.5) |
| 2004 | 22.4 (22.1 - 22.7) | 28.4 (27.9 - 28.8) |
| 2005 | 24.2 (23.9 - 24.5) | 29.9 (29.4 - 30.4) |
| 2006 | 23 (22.7 - 23.3) | 28.8 (28.4 - 29.2) |
| 2007 | 22.6 (22.3 - 22.9) | 28.2 (27.8 - 28.6) |
| 2008 | 23 (22.7 - 23.3) | 28.3 (27.8 - 28.7) |
| 2009 | 22.2 (21.9 - 22.5) | 28.2 (27.8 - 28.6) |
| 2010 | 22.7 (22.4 - 23) | 29.6 (29.2 - 30) |
| 2011 | 23.7 (23.4 - 24) | 30.4 (29.9 - 30.8) |
| 2012 | 24 (23.7 - 24.3) | 31.6 (31.1 - 32) |
| 2013 | 25.2 (24.9 - 25.5) | 32.9 (32.5 - 33.4) |
| 2014 | 25.7 (25.4 - 26) | 34.6 (34.2 - 35) |
| 2015 | 28.1 (27.8 - 28.4) | 37.5 (37 - 37.9) |
| 2016 | 28.3 (28 - 28.6) | 39.2 (38.8 - 39.6) |
| 2017 | 30.5 (30.2 - 30.9) | 42 (41.5 - 42.4) |
| 2018 | 32.1 (31.8 - 32.4) | 44.8 (44.3 - 45.2) |
| 2019 | 33.5 (33.2 - 33.8) | 47.4 (46.9 - 47.8) |
| 2020 | 36.8 (36.4 - 37.1) | 53.6 (53.2 - 54.1) |
| 2021 | 41.4 (41 - 41.8) | 60 (59.5 - 60.5) |
| 2022 | 40.5 (40.1 - 40.8) | 59.4 (58.9 - 59.9) |
| 2023 | 41.2 (40.9 - 41.6) | 59.7 (59.2 - 60.2) |

**Supplementary Table 4: Arrhythmias-related Age-Adjusted Mortality Rates per 100,000 in Adults with Heart Failure, Stratified by Race, in the United States, 1999 to 2020**

|  | **Age-Adjusted Mortality Rate (95% Confidence Interval)** | | | | | |
| --- | --- | --- | --- | --- | --- | --- |
| **Year** | **NH American Indian or Alaska Native** | **NH Black or African American** | | **NH Asian or Pacific Islander** | **NH White** | **Hispanic** |
| 1999 | 18.261 (14.545 - 22.637) | 22.625 (21.735 - 23.514) |  | 12.69 (11.272 - 14.109) | 26.35 (26.058 - 26.641) | 15.127 (14.091 - 16.163) |
| 2000 | 15.871 (12.525 - 19.837) | 21.039 (20.185 - 21.892) |  | 10.13 (8.91 - 11.351) | 26.065 (25.777 - 26.353) | 13.787 (12.828 - 14.746) |
| 2001 | 17.111 (13.609 - 21.239) | 21.331 (20.476 - 22.187) |  | 12.169 (10.904 - 13.434) | 26.64 (26.35 - 26.93) | 14.568 (13.609 - 15.526) |
| 2002 | 19.945 (16.011 - 23.88) | 21.719 (20.863 - 22.575) |  | 12.483 (11.235 - 13.731) | 26.608 (26.32 - 26.896) | 14.699 (13.756 - 15.643) |
| 2003 | 22.281 (18.156 - 26.407) | 21.901 (21.044 - 22.758) |  | 12.161 (10.962 - 13.361) | 26.765 (26.478 - 27.052) | 14.924 (14.002 - 15.847) |
| 2004 | 16.38 (13.119 - 20.204) | 21.234 (20.398 - 22.071) |  | 12.708 (11.524 - 13.892) | 26.006 (25.724 - 26.287) | 14.231 (13.351 - 15.111) |
| 2005 | 17.171 (13.7 - 20.642) | 22.865 (22.007 - 23.724) |  | 12.588 (11.463 - 13.712) | 27.786 (27.498 - 28.075) | 15.306 (14.418 - 16.195) |
| 2006 | 17.739 (14.294 - 21.184) | 20.909 (20.099 - 21.72) |  | 12.746 (11.647 - 13.845) | 26.73 (26.45 - 27.01) | 14.166 (13.337 - 14.995) |
| 2007 | 20.123 (16.429 - 23.816) | 20.582 (19.787 - 21.377) |  | 11.565 (10.553 - 12.578) | 26.295 (26.02 - 26.571) | 13.851 (13.054 - 14.647) |
| 2008 | 19.519 (15.959 - 23.079) | 20.731 (19.943 - 21.519) |  | 12.626 (11.595 - 13.656) | 26.646 (26.371 - 26.921) | 13.79 (13.018 - 14.562) |
| 2009 | 22.471 (18.748 - 26.193) | 19.717 (18.961 - 20.473) |  | 12.608 (11.612 - 13.604) | 26.202 (25.932 - 26.473) | 13.034 (12.309 - 13.76) |
| 2010 | 21.995 (18.364 - 25.626) | 20.324 (19.563 - 21.085) |  | 12.914 (11.93 - 13.898) | 27.181 (26.906 - 27.455) | 14.199 (13.456 - 14.943) |
| 2011 | 24.485 (20.764 - 28.206) | 20.915 (20.156 - 21.674) |  | 12.716 (11.787 - 13.645) | 28.405 (28.127 - 28.683) | 14.103 (13.396 - 14.811) |
| 2012 | 21.309 (18.001 - 24.617) | 22.013 (21.25 - 22.777) |  | 11.927 (11.062 - 12.792) | 29.092 (28.814 - 29.371) | 14.983 (14.277 - 15.689) |
| 2013 | 25.082 (21.612 - 28.553) | 22.452 (21.699 - 23.205) |  | 12.838 (11.977 - 13.698) | 30.646 (30.362 - 30.93) | 15.382 (14.688 - 16.077) |
| 2014 | 24.931 (21.536 - 28.327) | 23.911 (23.143 - 24.679) |  | 13.054 (12.219 - 13.89) | 31.82 (31.533 - 32.108) | 15.934 (15.252 - 16.615) |
| 2015 | 26.418 (23.087 - 29.749) | 25.346 (24.57 - 26.123) |  | 13.659 (12.835 - 14.482) | 34.742 (34.444 - 35.04) | 17.907 (17.207 - 18.606) |
| 2016 | 28.662 (25.259 - 32.066) | 25.412 (24.649 - 26.175) |  | 15.058 (14.215 - 15.901) | 35.805 (35.504 - 36.105) | 18.213 (17.526 - 18.901) |
| 2017 | 29.123 (25.755 - 32.492) | 28.227 (27.437 - 29.018) |  | 14.78 (13.974 - 15.586) | 38.706 (38.395 - 39.016) | 19.472 (18.78 - 20.164) |
| 2018 | 29.049 (25.86 - 32.237) | 29.453 (28.66 - 30.247) |  | 16.154 (15.335 - 16.973) | 41.157 (40.841 - 41.474) | 20.351 (19.662 - 21.04) |
| 2019 | 29.937 (26.734 - 33.139) | 30.837 (30.036 - 31.637) |  | 15.855 (15.065 - 16.645) | 43.481 (43.158 - 43.804) | 21.136 (20.448 - 21.824) |
| 2020 | 36.084 (32.684 - 39.485) | 37.112 (36.249 - 37.975) |  | 18.42 (17.593 - 19.246) | 48.229 (47.89 - 48.567) | 25.283 (24.548 - 26.018) |

**Supplementary Table 5: Arrhythmias-related Age-Adjusted Mortality Rates per 100,000 in Adults with Heart Failure, Stratified by Urban-Rural Classification, in the United States, 1999 to 2020**

| Year | **Metropolitan** | **Nonmetropolitan** |
| --- | --- | --- |
| 1999 | 24.538 (24.247 - 24.828) | 29.351 (28.704 - 29.998) |
| 2000 | 24.062 (23.776 - 24.348) | 28.608 (27.973 - 29.243) |
| 2001 | 24.566 (24.28 - 24.853) | 29.61 (28.966 - 30.253) |
| 2002 | 24.63 (24.346 - 24.914) | 29.534 (28.892 - 30.177) |
| 2003 | 24.535 (24.254 - 24.816) | 30.413 (29.765 - 31.061) |
| 2004 | 23.88 (23.605 - 24.155) | 29.141 (28.509 - 29.773) |
| 2005 | 25.504 (25.223 - 25.785) | 31.082 (30.433 - 31.732) |
| 2006 | 24.444 (24.172 - 24.716) | 29.859 (29.227 - 30.49) |
| 2007 | 24.017 (23.751 - 24.283) | 28.97 (28.352 - 29.589) |
| 2008 | 24.161 (23.897 - 24.425) | 30.135 (29.507 - 30.762) |
| 2009 | 23.65 (23.391 - 23.908) | 29.664 (29.045 - 30.284) |
| 2010 | 24.541 (24.279 - 24.802) | 30.354 (29.731 - 30.977) |
| 2011 | 25.608 (25.345 - 25.871) | 30.77 (30.15 - 31.391) |
| 2012 | 26.124 (25.862 - 26.387) | 32.217 (31.586 - 32.849) |
| 2013 | 27.472 (27.206 - 27.737) | 33.186 (32.551 - 33.822) |
| 2014 | 28.361 (28.094 - 28.628) | 35.06 (34.41 - 35.711) |
| 2015 | 30.889 (30.613 - 31.164) | 38.075 (37.402 - 38.748) |
| 2016 | 31.742 (31.466 - 32.019) | 38.906 (38.23 - 39.582) |
| 2017 | 34.047 (33.765 - 34.33) | 42.494 (41.793 - 43.195) |
| 2018 | 35.894 (35.608 - 36.18) | 45.817 (45.096 - 46.538) |
| 2019 | 37.535 (37.245 - 37.825) | 49.194 (48.451 - 49.938) |
| 2020 | 41.975 (41.672 - 42.278) | 54.771 (53.991 - 55.551) |

**Supplementary Table 6: Arrhythmias-related Age-Adjusted Mortality Rates per 100,000 in Adults with Heart Failure, Stratified by Census Regions, in the United States, 1999 to 2023**

| **Year** | **Northeast** | **Midwest** | **South** | **West** |
| --- | --- | --- | --- | --- |
| 1999 | 23.6 (23 - 24.1) | 28.5 (28 - 29.1) | 23.8 (23.4 - 24.2) | 26.6 (26 - 27.2) |
| 2000 | 23.2 (22.7 - 23.8) | 28 (27.5 - 28.6) | 23.4 (23 - 23.8) | 25.8 (25.2 - 26.4) |
| 2001 | 23.5 (22.9 - 24) | 28.6 (28 - 29.2) | 24 (23.6 - 24.4) | 26.6 (26 - 27.2) |
| 2002 | 22.6 (22 - 23.1) | 29.1 (28.5 - 29.6) | 24 (23.5 - 24.4) | 27.2 (26.6 - 27.8) |
| 2003 | 22.5 (22 - 23) | 28.4 (27.8 - 28.9) | 24.5 (24 - 24.9) | 27.7 (27.1 - 28.3) |
| 2004 | 22.4 (21.9 - 22.9) | 27.3 (26.7 - 27.8) | 22.9 (22.5 - 23.3) | 27.9 (27.3 - 28.5) |
| 2005 | 23.2 (22.7 - 23.8) | 29.5 (28.9 - 30.1) | 25 (24.6 - 25.4) | 29 (28.4 - 29.6) |
| 2006 | 22.3 (21.8 - 22.8) | 27.8 (27.3 - 28.3) | 23.8 (23.4 - 24.2) | 28.5 (28 - 29.1) |
| 2007 | 21.8 (21.3 - 22.4) | 27 (26.5 - 27.5) | 23.5 (23.1 - 23.9) | 27.9 (27.4 - 28.5) |
| 2008 | 22.5 (22 - 23) | 27.9 (27.4 - 28.4) | 23 (22.6 - 23.4) | 28.5 (27.9 - 29.1) |
| 2009 | 22.1 (21.6 - 22.6) | 26.3 (25.8 - 26.9) | 23 (22.6 - 23.4) | 28.2 (27.6 - 28.7) |
| 2010 | 23.3 (22.8 - 23.8) | 27.1 (26.6 - 27.7) | 23.8 (23.4 - 24.2) | 28.9 (28.3 - 29.5) |
| 2011 | 24.2 (23.7 - 24.7) | 29 (28.5 - 29.6) | 23.9 (23.5 - 24.3) | 30.3 (29.7 - 30.9) |
| 2012 | 24.8 (24.3 - 25.3) | 29.6 (29 - 30.1) | 25.2 (24.8 - 25.6) | 30.1 (29.6 - 30.7) |
| 2013 | 26.2 (25.7 - 26.7) | 30.5 (30 - 31) | 26.3 (25.9 - 26.7) | 31.9 (31.4 - 32.5) |
| 2014 | 27.1 (26.6 - 27.6) | 31.9 (31.4 - 32.5) | 27.5 (27.2 - 27.9) | 32.3 (31.8 - 32.9) |
| 2015 | 28.9 (28.3 - 29.4) | 35.2 (34.7 - 35.8) | 29.7 (29.3 - 30.1) | 35.7 (35.1 - 36.2) |
| 2016 | 28.8 (28.3 - 29.4) | 35 (34.4 - 35.6) | 30.8 (30.4 - 31.2) | 37.8 (37.2 - 38.4) |
| 2017 | 30.5 (29.9 - 31) | 38.8 (38.2 - 39.4) | 33.5 (33.1 - 33.9) | 39.5 (38.9 - 40.1) |
| 2018 | 32.4 (31.9 - 33) | 42 (41.4 - 42.6) | 35.5 (35.1 - 36) | 40.6 (40 - 41.2) |
| 2019 | 33.3 (32.7 - 33.8) | 43.9 (43.3 - 44.5) | 38.3 (37.8 - 38.7) | 42.2 (41.6 - 42.8) |
| 2020 | 38.7 (38.1 - 39.3) | 49.7 (49 - 50.3) | 42.2 (41.7 - 42.6) | 46.1 (45.5 - 46.7) |
| 2021 | 41.1 (40.5 - 41.8) | 54.7 (54 - 55.4) | 48.2 (47.7 - 48.7) | 53.4 (52.7 - 54.1) |
| 2022 | 40.4 (39.7 - 41) | 53 (52.3 - 53.6) | 48 (47.5 - 48.5) | 51.9 (51.3 - 52.6) |
| 2023 | 41.1 (40.5 - 41.7) | 54.5 (53.9 - 55.2) | 48.7 (48.2 - 49.2) | 51.6 (51 - 52.3) |

**Supplementary Table 7: Arrhythmias-related Age-Adjusted Mortality Rates per 100,000 in Adults with Heart Failure, Stratified by State, in the United States, 1999 to 2023**

| **State** | **Age-adjusted Mortality Rate (95% Confidence Interval)** | |
| --- | --- | --- |
|  | 1999-2020 | 2021-2023 |
| Alabama | 26.266 (25.845 - 26.687) | 34.838 (33.65 - 36.026) |
| Alaska | 31.433 (29.669 - 33.198) | 39.54 (35.268 - 43.812) |
| Arizona | 19.359 (19.049 - 19.67) | 37.639 (36.651 - 38.626) |
| Arkansas | 30.157 (29.597 - 30.717) | 52.645 (50.773 - 54.516) |
| California | 32.931 (32.754 - 33.108) | 45.161 (44.659 - 45.663) |
| Colorado | 35.25 (34.725 - 35.775) | 73.021 (71.254 - 74.787) |
| Connecticut | 26.913 (26.465 - 27.361) | 30.278 (29.043 - 31.513) |
| Delaware | 27.807 (26.833 - 28.781) | 49.513 (46.47 - 52.556) |
| District of Columbia | 21.259 (20.157 - 22.361) | 33.282 (29.759 - 36.805) |
| Florida | 17.831 (17.681 - 17.98) | 35.269 (34.771 - 35.768) |
| Georgia | 21.838 (21.529 - 22.147) | 35.019 (34.118 - 35.921) |
| Hawaii | 19.932 (19.293 - 20.571) | 30.141 (28.3 - 31.983) |
| Idaho | 38.483 (37.558 - 39.407) | 75.972 (72.948 - 78.995) |
| Illinois | 25.25 (24.999 - 25.5) | 35.743 (34.985 - 36.5) |
| Indiana | 35.419 (35.002 - 35.837) | 59.419 (58.055 - 60.784) |
| Iowa | 32.309 (31.791 - 32.827) | 64.141 (62.209 - 66.073) |
| Kansas | 28.277 (27.739 - 28.814) | 49.044 (47.204 - 50.884) |
| Kentucky | 34.535 (34.022 - 35.048) | 62.748 (61.026 - 64.47) |
| Louisiana | 23.288 (22.865 - 23.711) | 44.931 (43.463 - 46.4) |
| Maine | 30.913 (30.135 - 31.691) | 53.196 (50.668 - 55.724) |
| Maryland | 28.583 (28.174 - 28.992) | 54.958 (53.599 - 56.317) |
| Massachusetts | 24.672 (24.35 - 24.995) | 41.962 (40.889 - 43.034) |
| Michigan | 28.841 (28.546 - 29.136) | 45.076 (44.133 - 46.02) |
| Minnesota | 40.34 (39.864 - 40.816) | 84.167 (82.456 - 85.879) |
| Mississippi | 30.371 (29.781 - 30.96) | 61.835 (59.715 - 63.954) |
| Missouri | 27.521 (27.153 - 27.889) | 44.278 (43.092 - 45.465) |
| Montana | 31.091 (30.149 - 32.034) | 52.427 (49.421 - 55.432) |
| Nebraska | 35.877 (35.132 - 36.623) | 80.084 (77.191 - 82.976) |
| Nevada | 16.739 (16.231 - 17.248) | 39.223 (37.517 - 40.929) |
| New Hampshire | 33.309 (32.433 - 34.186) | 54.208 (51.528 - 56.888) |
| New Jersey | 25.025 (24.737 - 25.314) | 35.099 (34.242 - 35.957) |
| New Mexico | 23.037 (22.422 - 23.653) | 31.878 (30.158 - 33.598) |
| New York | 20.832 (20.655 - 21.009) | 32.785 (32.231 - 33.338) |
| North Carolina | 31.994 (31.656 - 32.333) | 51.349 (50.332 - 52.366) |
| North Dakota | 40.127 (38.898 - 41.356) | 59.192 (55.308 - 63.075) |
| Ohio | 38.859 (38.546 - 39.172) | 51.793 (50.864 - 52.722) |
| Oklahoma | 39.156 (38.579 - 39.733) | 76.331 (74.287 - 78.375) |
| Oregon | 47.106 (46.506 - 47.706) | 102.197 (100.007 - 104.386) |
| Pennsylvania | 31.389 (31.139 - 31.639) | 53.227 (52.378 - 54.075) |
| Rhode Island | 39.733 (38.75 - 40.716) | 54.519 (51.523 - 57.514) |
| South Carolina | 35.02 (34.517 - 35.523) | 63.38 (61.805 - 64.956) |
| South Dakota | 33.983 (32.938 - 35.028) | 71.147 (67.191 - 75.103) |
| Tennessee | 35.337 (34.907 - 35.767) | 56.254 (54.932 - 57.576) |
| Texas | 34.329 (34.089 - 34.569) | 53.298 (52.595 - 54.001) |
| Utah | 31.798 (31.052 - 32.543) | 55.179 (52.859 - 57.499) |
| Vermont | 44.124 (42.709 - 45.538) | 69.97 (65.598 - 74.342) |
| Virginia | 25.309 (24.975 - 25.644) | 54.078 (52.918 - 55.238) |
| Washington | 45.1 (44.626 - 45.574) | 72.034 (70.592 - 73.477) |
| West Virginia | 40.584 (39.811 - 41.357) | 54.859 (52.521 - 57.198) |
| Wisconsin | 33.273 (32.863 - 33.683) | 71.905 (70.36 - 73.45) |
| Wyoming | 32.449 (31.033 - 33.865) | 68.449 (63.458 - 73.44) |

**Supplementary Table 8: Overall Heart Failure vs Arrhythmias-related Age-Adjusted Mortality Rates per 100,000 in Adults with Heart Failure in the United States, 1999 to 2023**

|  | AAMR (95% Confidence Interval) | |
| --- | --- | --- |
| Year | Overall Heart Failure | Overall Heart Failure and Arrhythmias |
| 1999 | 212.003 (211.236 - 212.769) | 25.5 (25.2 - 25.7) |
| 2000 | 209.24 (208.484 - 209.996) | 24.9 (24.7 - 25.2) |
| 2001 | 204.038 (203.297 - 204.778) | 25.5 (25.3 - 25.8) |
| 2002 | 200.323 (199.594 - 201.051) | 25.5 (25.3 - 25.8) |
| 2003 | 198.805 (198.085 - 199.525) | 25.7 (25.4 - 25.9) |
| 2004 | 192.561 (191.857 - 193.264) | 24.8 (24.6 - 25.1) |
| 2005 | 193.602 (192.904 - 194.3) | 26.5 (26.3 - 26.8) |
| 2006 | 183.195 (182.523 - 183.867) | 25.4 (25.2 - 25.7) |
| 2007 | 175.683 (175.032 - 176.334) | 24.9 (24.7 - 25.2) |
| 2008 | 174.628 (173.985 - 175.27) | 25.2 (25 - 25.5) |
| 2009 | 166.92 (166.298 - 167.543) | 24.7 (24.5 - 25) |
| 2010 | 166.788 (166.171 - 167.406) | 25.6 (25.3 - 25.8) |
| 2011 | 164.563 (163.958 - 165.168) | 26.5 (26.3 - 26.8) |
| 2012 | 161.59 (160.997 - 162.182) | 27.2 (26.9 - 27.4) |
| 2013 | 165.48 (164.887 - 166.073) | 28.5 (28.2 - 28.7) |
| 2014 | 166.762 (166.173 - 167.351) | 29.5 (29.3 - 29.8) |
| 2015 | 174.739 (174.142 - 175.335) | 32.1 (31.8 - 32.3) |
| 2016 | 174.72 (174.129 - 175.311) | 32.9 (32.7 - 33.2) |
| 2017 | 180.022 (179.429 - 180.615) | 35.5 (35.2 - 35.7) |
| 2018 | 184.291 (183.699 - 184.884) | 37.5 (37.3 - 37.8) |
| 2019 | 187.371 (186.779 - 187.962) | 39.5 (39.2 - 39.7) |
| 2020 | 204.835 (204.222 - 205.449) | 44.1 (43.8 - 44.4) |
| 2021 | 218.683 (218.033 - 219.332) | 49.4 (49.1 - 49.8) |
| 2022 | 211.491 (210.872 - 212.11) | 48.5 (48.2 - 48.8) |
| 2023 | 210.333 (209.715 - 210.952) | 49.2 (48.9 - 49.5) |

**Supplementary Table 9: Arrhythmias-related Deaths in Adults with Heart Failure, Stratified by Place of Death, in the United States, 1999 to 2023**

|  | **Deaths** | | | | | |
| --- | --- | --- | --- | --- | --- | --- |
| **Year** | **Medical facility** | **Decedent’s Home** | **Hospice Facility** | **Nursing home/long term care** | **Other** | **Place of death unknown** |
| 1999 | 17902 | 6231 | - | 10327 | 823 | - |
| 2000 | 17341 | 6256 | - | 10544 | 922 | 17 |
| 2001 | 17800 | 6543 | - | 11066 | 1043 | - |
| 2002 | 17670 | 6977 | - | 11161 | 1194 | - |
| 2003 | 17938 | 7232 | 46 | 11227 | 1265 | 90 |
| 2004 | 17170 | 7371 | 99 | 11065 | 1382 | 94 |
| 2005 | 18455 | 7953 | 374 | 12207 | 1432 | 113 |
| 2006 | 17653 | 8249 | 564 | 11784 | 1370 | 143 |
| 2007 | 17274 | 8479 | 838 | 11738 | 1423 | 111 |
| 2008 | 17661 | 8711 | 1024 | 11942 | 1450 | 454 |
| 2009 | 16823 | 9221 | 1048 | 11827 | 1662 | 701 |
| 2010 | 17559 | 10164 | 1453 | 12440 | 1815 | 24 |
| 2011 | 18492 | 11051 | 1731 | 13266 | 1986 | 16 |
| 2012 | 18510 | 12118 | 2253 | 13795 | 2176 | 28 |
| 2013 | 19593 | 13240 | 2384 | 14745 | 2479 | 25 |
| 2014 | 20260 | 14711 | 2811 | 15435 | 2298 | 36 |
| 2015 | 22151 | 16585 | 3620 | 16932 | 2488 | 15 |
| 2016 | 22996 | 17925 | 4004 | 17048 | 2701 | 10 |
| 2017 | 24885 | 19836 | 4694 | 18651 | 3055 | - |
| 2018 | 27150 | 21870 | 5254 | 19563 | 3251 | 15 |
| 2019 | 28558 | 23759 | 5981 | 20650 | 3544 | 13 |
| 2020 | 32116 | 31243 | 5806 | 20411 | 4263 | 21 |
| 2021 | 36481 | 33154 | 6470 | 19315 | 4486 | 10 |
| 2022 | 38242 | 33252 | 6704 | 21036 | 5102 | - |
| 2023 | 37664 | 32825 | 7330 | 22843 | 4557 | - |
| Total | 558344 | 374956 | 64488 | 371018 | 58167 | 1936 |

**Figures:**


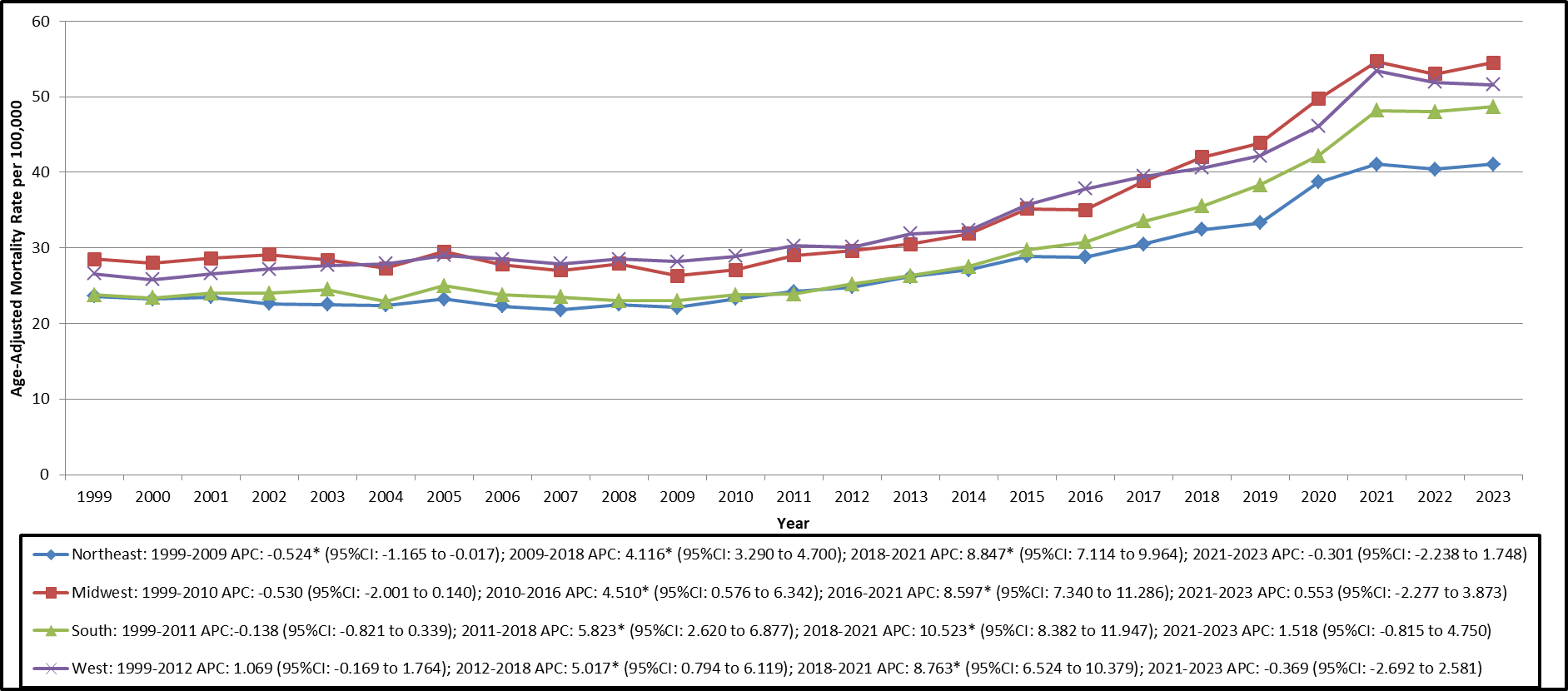


***Supplementary Figure 1:* Trends in Arrhythmias related Age-Adjusted Mortality rates per 100,000 in Adults with Heart Failure in the United States, Stratified by Census Regions, from 1999 to 2023**


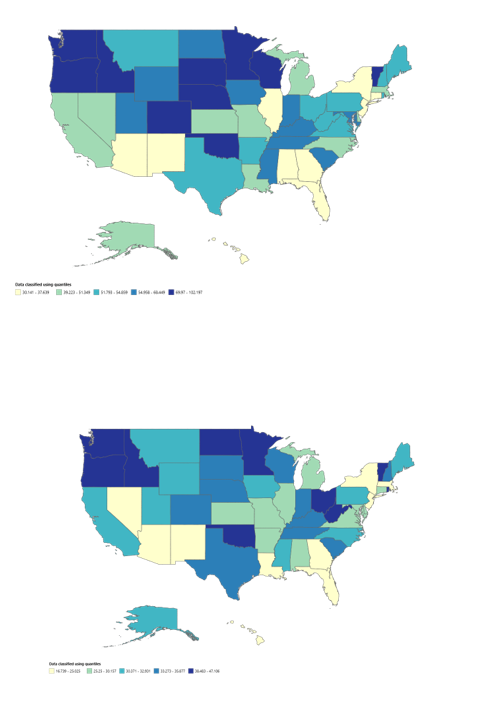


***Supplementary Figure 2:* Trends in Arrhythmias related Age-Adjusted Mortality rates per 100,000 in Adults with Heart Failure in the United States, Stratified by States, from 1999 to 2023**

A-1999 to 2020

B- 2021-2023
